# Time to Rethink Intended Learning Outcomes for Sustainable Development? A Qualitative Exploration and Reflection of Course Syllabuses in Swedish Undergraduate Physiotherapy Education

**DOI:** 10.1177/23821205241260599

**Published:** 2024-06-05

**Authors:** Emma Swärdh, Nina Brodin, Anna Pettersson, Annie Palstam

**Affiliations:** 1 Division of Physiotherapy, Department of Neurobiology, Health Care Sciences and Society, 27106 Karolinska Institutet, Huddinge, Sweden; 2 Division of Physiotherapy, Department of Orthopaedics, Danderyd Hospital, Stockholm, Sweden; 3 101092 School of Health and Welfare, Dalarna University, Falun, Sweden; 4 Department of Clinical Neuroscience, Institute of Neuroscience and Physiology, Sahlgrenska Academy, 70712 University of Gothenburg, Gothenburg, Sweden

**Keywords:** Higher education, sustainable development, syllabuses undergraduate physiotherapy education

## Abstract

**OBJECTIVE:**

Several calls to action for the implementation of education for sustainable development in health profession education have been put forth during the last few years. The aim was therefore to explore and describe sustainability-focused intended learning outcomes (SD-ILOs) in curricula of undergraduate physiotherapy education in Sweden.

**METHODS:**

Using a deductive, descriptive, and qualitative approach, SD-ILOs in programs (*n* = 8) and course syllabuses (*n* = 143) from eight higher education institutions providing physiotherapy undergraduate education in Sweden were analyzed. SD-ILOs were described based on the subject content or condition, level of cognitive processes, sustainability learning dimensions, and key sustainability competencies.

**RESULTS:**

Six of the eight physiotherapy programs provided course syllabuses with SD-ILOs. However, only 3% (*n* = 36) of all ILOs were sustainability-focused. A larger part of the SD-ILOs, 78% (*n* = 28) was described within the cognitive dimension of learning, and 80% (*n* = 27) were linked to either the cognitive process ‘understanding’ or ‘analyzing’. The most frequently identified key competency in the SD-ILOs was ‘systems-thinking’ *n* = 10 (28%), and 30% (*n* = 11) lacked key competency.

**CONCLUSION:**

There is an urgent need for rapid initiatives to enhance sustainable development education in Swedish undergraduate physiotherapy education. Pedagogical approaches that cover not only cognitive dimensions of learning for sustainable development but also socio-emotional and behavioral dimensions, as well as more complex cognitive learning processes must also be developed. The current lack of key sustainability competencies further emphasizes the necessity to enrich physiotherapy curricula with action-oriented learning to develop powerful future sustainability agency within healthcare and the public health arena.

## Introduction

The concept of sustainable development emerged in the latter half of the 20^th^ century and was officially introduced and put on the international agenda by the United Nations World Commission for Environment and Development in 1987.^1^ It originally focused on minimizing harm to the environment, but it has since then evolved into a broader understanding that encompasses three key domains: ecological-, economic- and social sustainability.^2^ This entails preserving the earth's natural resources, promoting social progress, and fostering economic growth with consideration of future generations’ ability to meet their own needs. Education for Sustainable Development (ESD) is internationally recognized as a vital component of sustainable development.^3^ The essentiality of learners to acquire, develop, and apply necessary knowledge and skills for sustainable development has been emphasized by the United Nations Decade of Education for Sustainable Development since 2005 and is also highlighted in the Sustainable Development Goals (SDG), particularly SDG 4.7.^4^ Education for sustainable development is defined as education that aims to prepare people to cope with and find solutions, acknowledging the co-dependence between all three domains of sustainable development.^5^ Thus, it is important to integrate ESD in Higher Education (HE) to equip students with the ability to think and act in line with sustainability principles in order to contribute to a sustainable society.^6^ This requires educational approaches based on paradigms within sustainable development. However, the understanding of sustainable development and its implementation in HE is continuously changing as a result of shifts in epistemology.^7,8^

A consensus statement by the International Association for Health Professions Education highlights the need for reforms in education that equip future healthcare professionals with the knowledge and abilities to provide sustainable healthcare with a focus on the ecological domain of sustainable development.^9^ Voices have also been raised to integrate sustainable development within physiotherapy.^10–14^ Since health professionals are often referred to as trusted members of society,^15^ it becomes essential to accelerate the involvement of educators within health professions in delivering education that advocates for sustainable development. Since 2006, the initial provisions of the Swedish Higher Education Act state that HE should promote sustainability in their activities.^16^ However, in 2017, the Swedish Higher Education Authority (UKÄ) which monitors compliance with laws and regulations found that six of eight universities providing undergraduate physiotherapy education need development in their work on sustainable development within education.^17,18^ Furthermore, the evaluation showed that some Higher Education Institutions (HEIs) are uncertain about the meaning of the term ‘sustainable development’, and thus highlighted the importance of an understanding of the global goals for sustainable development, as universal, indivisible, and linked. Yet, the qualification descriptor for Bachelor of Science in Physiotherapy at the first cycle level in Sweden does not explicitly include objectives on sustainable development.^19^

Evaluating the extent to which sustainable development is incorporated into curricula serves as an initial step toward change. In outcome-based education, decisions regarding the course content, teaching and learning activities, and assessments are guided by the intended learning outcomes (ILOs).^20^ The concept of an ILO can be defined in various ways based on differing perspectives on learning; however, they generally serve as a foundation for designing courses and informing educators and students about the intellectual or practical skills that students need to acquire, develop, and apply.^21^ For learning of ESD to be meaningful, a slightly different way of thinking when it comes to designing ILOs is required. Typically, an ILO would consist of three elements: an action verb, a content, and a condition to provide the context. The action verb generally refers to the intended cognitive process that can be classified into levels of increasing complexity. ILOs are then typically divided into three categories of learning: cognitive, affective, or psychomotor dimensions.^22^ In learning of ESD, however, it is important to categorize ILOs in terms of cognitive-, socioemotional-, and behavioral dimensions of learning and also to develop these simultaneously. These dimensions are indispensable for a value-oriented and holistic approach in ESD, and an overlying emphasis on for example cognitive dimension of learning may result in learners who are less inclined to modify their daily behaviors and actively participate in creating a more inclusive, equitable and sustainable society.^23^ In addition, there is a set of sustainability competencies that should be developed through ESD to enable students to deal with unique and complex problems that lack definitive formulations, inherent logic, and solutions, as well as to engage with sustainability-related issues.^24^ Thus, this highlights the need to rethink the ILO within HE with regard to ESD.

Several studies have focused on different aspects of ESD curricula in HE for healthcare professions.^25–28^ Yet to the best of our knowledge, no research has been done on course syllabuses in undergraduate physiotherapy education that reflect ILOs, levels of cognitive processes, sustainability learning dimensions, and key sustainability competencies. The purpose of this study is to explore and describe the integration of sustainable development in the program and course syllabuses of undergraduate physiotherapy education in Sweden.

Research questions:

- To what extent is sustainable development addressed in ILOs in local Swedish undergraduate physiotherapy programs and course syllabuses?
- How is sustainable development expressed in the ILOs?
- Which level of cognitive processes, sustainability learning dimensions, and key sustainability competencies are in focus in the ILOs addressing sustainable development?

## Material and methods

### Research design

This research has been undertaken within the framework of a deductive, descriptive, and qualitative approach.^29^ The reporting of this study conforms to the Standards for Reporting Qualitative Research (SRQR)^30^ [Supplementary File].

### Context and data collection

The Swedish national study program in physiotherapy at the first cycle level (as part of the adaptation to the Bologna Process) comprises 180 European Credit Transfer and Accumulation System credits (ECTS) or three years of full-time studies and is provided by eight different HEIs geographically spread over the country. The education follows the qualification descriptor for Bachelor of Science in Physiotherapy.^19^ In addition, each HEI can adopt local regulations and requirements. A program syllabus for each local study program and a course syllabus for each course within the program is required. The Swedish version of a course syllabus is a legally binding document. As per Swedish law, each course syllabus must outline the title of the course, the number of higher education credits, its level, ILOs, main content, specific entry requirements, how the student performance is assessed, and what course literature is required or relevant. Thus, all Swedish course syllabuses from year one to three in eight physiotherapy programs in Sweden, valid in autumn 2023, were collected from the HEIs local web pages or through personal contact with the HEIs.

### Ethics

It was not deemed necessary to apply for ethical review from the Swedish Ethical Review Authority, as no intervention was made, the data were collected from public domains and no processing of sensitive data took place.

### Data analysis

The data used as units for the analysis of the course syllabus was the formulation of the ILOs. The data were analyzed using manifest deductive content analysis.^31,32^ The course syllabuses were analyzed by reading documents, coding and categorizing ILOs, and calculating frequencies and percentages of categories (Table 1). First, the analysis focused on extracting intended learning outcomes including sustainable development (SD-ILOs) from the syllabuses. The chosen SD-ILOs were then deductively mapped by the level of cognitive processes, sustainability learning dimension, as well as the type of key sustainability competencies from the worded SD-ILOs. To ensure trustworthiness, all members of the research team took part in the analysis. The researchers were physiotherapists and teachers within undergraduate and graduate physiotherapy education with extensive experience in writing, reading, and analyzing program and course syllabuses. All the researchers had expertise in qualitative research approaches. Two of the researchers (ES, APa) also had deep knowledge in sustainable development, and one of the researchers (Ape), in medical education. To harmonize the understanding and interpretation of data, multiple consultations within the research team were performed before the analysis was started. A study protocol was developed and used during the analysis describing the theoretical framework and definitions on which the deductive analysis was based. The researchers also engaged in team discussions throughout the research process, where thoughts and existing assumptions were analyzed and challenged. Further, all members of the research team participated in the discussion of the result. The final data were extracted from each university's web page in July 2023 and the analysis was performed during August-November 2023.

**Table 1.** The steps of the deductive data analysis. The number in brackets represents the theoretical framework in each step.

To familiarize and get an overview of the manifest material, all local program and course syllabuses were read carefully by one of the authors (ES). The author (ES) then selected ILOs to be further analyzed according to the criteria for SD-ILOs [1]. During this phase of the analysis, the SD-ILOs were discussed with a second author (APa) until a consensus was met. Open coding was then performed with all the material through writing notes (words, sentences, and paragraphs) in the chosen SD-ILOs, using a deductive approach. The material was read through several times while related parts were identified. The SD-ILOs were then moved/collected from the syllabuses into a separate analyzing sheet. Two of the researchers (ES and APe) analyzed the SD-ILOs independently, labeled them with codes, sorted and abstracted them into categories. Deductive coding was used to assign an appropriate categorization for the SD-ILOs into sustainability as subject content or a condition [2], level of cognitive process [3], learning dimensions [4], and key competencies [5]. Any differences between the researchers (ES and APe) in the data interpretation were discussed until a negotiated consensus was reached. Frequencies and percentages were calculated to describe the total number of local program and course syllabuses with SD-ILOs.

### Theoretical framework for the deductive approach

To detect sustainability content in the ILOs and to define sustainable development as content or context, level of cognitive processes, sustainability learning dimension, and key competencies to the ILOs, we used the following theoretical frameworks:

#### 1. Learning outcomes with sustainable development

The analysis of the ILOs in the course syllabuses was performed starting from the criteria defined by the Sustainability Tracking, Assessment & Rating System (STAR), developed by the Association for the Advancement of Sustainability in Higher Education.^33^ According to those guidelines, sustainability-focused ILOs are student ILOs that explicitly address the concept of sustainability. An ILO does not necessarily have to include the term ‘sustainability’ to count as sustainability-focused if there is an explicit focus on the interdependence of ecological systems and social/economic systems. Thus, all ILOs explicitly including the wording of sustainable development, sustainability perspective, UN sustainable development goals, global sustainable development goals, or sustainable were included. ILOs that could be critical for addressing sustainability challenges but not explicitly addressing the concept of sustainability, or explicitly focusing on only one of the three sustainability dimensions were not included in the analysis.

#### 2. Sustainable development as content or condition in learning outcomes

The content in an ILO represents the topic knowledge/values/skills to be demonstrated through a behavior (the action verb). The condition is the setting or circumstances under which the behavior and learning will occur.^20^

#### 3. Level of cognitive processes

Bloom's revised taxonomy of the cognitive domain classifies the cognitive processes in learning into six levels of increasing complexity.^22^ Usually, the action verb in the ILO relates to the cognitive process. The six levels are: *remembering;* memorize, retrieve, recall, or recognize basic facts, dates, events, persons, places, concepts, and patterns, *understanding;* demonstrate or explain facts, concepts, or ideas in own words by organizing and comparing, *applying;* use learned facts, information, and abstractions in new contexts and particular situations, *analyzing;* break down concepts and examine how the parts relate to one another and/or to an overall structure or purpose, *evaluating;* appraise a situation by making a judgment about information based on criteria and standards, *creating;* combine known elements, patterns, ideas and facts to form new original work or formulate their solution to a problem.

#### 4. Sustainability learning dimensions

Sustainability-focused intended learning outcomes can be classified according to the emphasis on a cognitive, socio-emotional, or behavioral dimension of learning.^23^ *The cognitive learning dimension* comprises knowledge and critical thinking skills necessary to understand sustainable development challenges and their complex interconnectedness and to explore alternative solutions. The *social and emotional learning dimension* includes skills to build core, values and attitudes for sustainable development, such as self-reflection skills as well as cultivating empathy and compassion for other people and the planet, as well as to motivate to lead change. Finally, the *behavioral learning dimension* describes skills to take effective and responsible action for a sustainable world in the personal, societal, and political spheres.

#### 5. Key sustainability competencies

A number of key sustainability competencies have been described and considered as necessary for learning related to sustainable development. It is now recognized that systems-, strategic-, futures-, values-thinking, intrapersonal-, implementation-, and interpersonal competencies collectively can contribute to an integrated problem-solving competency for sustainability^24^ (Table 2).

**Table 2.** Definitions of key sustainability competencies.^24^

**Competency**	
**Systems-thinking**	identify and comprehend connections analyze intricate systems recognize how these systems are interconnected with various domains, and effectively navigate through uncertainty
**Future-thinking**	continuously refine and expand one's visions and scenarios challenge existing assumptions about how society operates reflect on assumptions and gain a better understanding of their influence on the approach to envisioning future possibilities
**Values-thinking**	recognize intrinsic and extrinsic values in social and natural contexts acknowledge oppressive systems and clarify personal values understand contextual influences on reinforcement critically evaluate alignment with sustainability principles and distinguish advocated from practiced values
**Strategic-thinking**	identify the historical origins and lasting resistance of intentional and unintended unsustainable practices as well as the obstacles to change plan to create experiments to test new strategies
**Intrapersonal competency**	critically reflect on one's own engagement in the local community and global society constantly evaluate and enhance one's actions effectively manage emotions and desires
**Implementation competency**	execute a planned solution that aligns with a sustainability-informed vision monitor and evaluate the progress of the implementation process and effectively address any unforeseen obstacles acknowledge that solving sustainability problems is an ongoing, cyclical journey involving planning, execution, and evaluation phases
**Interpersonal competency**	apply the concepts and methods of each competency in a way that effectively motivates and engages diverse stakeholders empathetic collaboration with partners and citizens, considering their unique perspectives, knowledge, and modes of communication
**Integrated problem-solving competency**	effectively combine and integrate various steps of the sustainability problem-solving process utilize relevant disciplinary, interdisciplinary, transdisciplinary, and other forms of knowledge

## Result

### Local program syllabuses

Of the eight local program syllabuses, one included a SD-ILO; ‘be able to work for a sustainable and health-promoting development of current and future generations’ and one had an overall objective for the education; ‘contribute to the understanding and promotion of sustainable development ensuring a healthy and sound environment, economic and social well-being and equity for present and future generations’.

### Local course syllabuses

#### Extent of learning outcomes addressing sustainable development

Six of the eight physiotherapy programs (75%) provided course syllabuses with SD-ILOs. Of the 143 local course syllabuses, 28 (20%) courses included one or more SD-ILOs. Further, of the 1255 ILOs within the programs, 36 (3%) were sustainability-focused. There was a variation in the number of SD-ILOs between the eight HEIs (Table 3). SD-ILOs were found in courses in all years, however the majority were within the first year. Year one had 19 (53%), year two had eight (22%), and year three had nine (25%). Only three programs had SD-ILOs during all three years.

**Table 3.** Overview of each HEI percentage of sustainability-focused intended learning outcomes (SD-ILOs) of the total intended learning outcomes (ILOs) in the program.

Higher institution	Number of ILO, *n*	SD-ILO, *n* (%)
**1**	135	7 (5)
**2**	130	1 (0,7)
**3**	234	8 (3)
**4**	103	0 (0)
**5**	152	1 (0,7)
**6**	212	16 (8)
**7**	150	0 (0)
**8**	139	3 (2)

**Figure 1.**
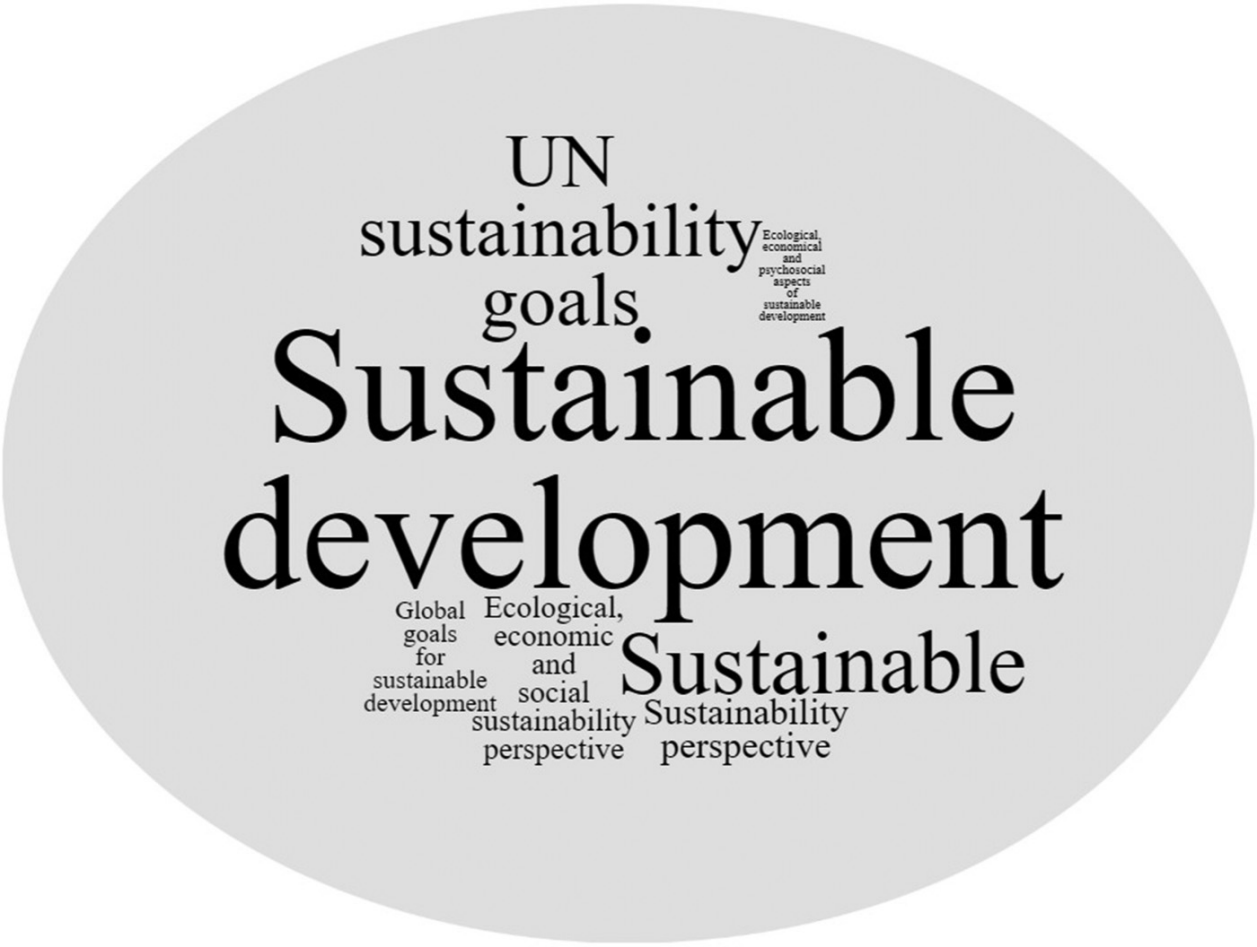
Overview of terms connected to sustainability in the learning outcomes.

The most frequently used term in the SD-ILOs was ‘sustainable development’ (13 times) (Figure 1). Other terms used were ‘sustainable’ (7 times), ‘UN sustainability goals’ (6 times), ‘ecological, economic and social sustainability perspective’ (5 times), ‘sustainability perspective’ (3 times), ‘global goals for sustainable development’ (2 times), and ‘ecological, economical and psychosocial aspects of sustainable development’ (1 time).

#### Sustainable development as content or condition

About half of the SD-ILOs *n* = 17 (47%) addressed sustainability as content and the other half as a condition *n* = 19 (50%) (Table 4).

**Table 4.** Examples of sustainable development as content or a condition.

**Content**	Reflect on how *sustainable development* can be included in physiotherapy research based on your own values and the literature. Reflect on *sustainable development* in relation to physiotherapy practice. Reflect on the concept of *sustainable development* and identify possibilities and challenges in achieving *the global goals for sustainable development* within the profession of physiotherapy
**Conditions**	Discuss physiotherapy hypotheses and research *from an equality and sustainability perspective*. Discuss physiotherapy *in a globally ecological, economic, and social sustainability perspective*. Evaluate and reflect on the role of the physiotherapist in achieving national and global goals (2030) in public health and *sustainable development*.

#### Level of cognitive processes

A total of 13 (36%) and 14 (44%) respectively of the SD-ILOs were linked to either the cognitive process of ‘understanding’ or ‘analyzing’. Other cognitive processes were ‘remembering’ *n* = 3 (8%), ‘applying’ *n* = 1 (3%), ‘evaluating’ *n* = 2 (6%), and ‘creating’ *n* = 1 (3%).

#### Sustainability learning dimensions

**Figure 2.**
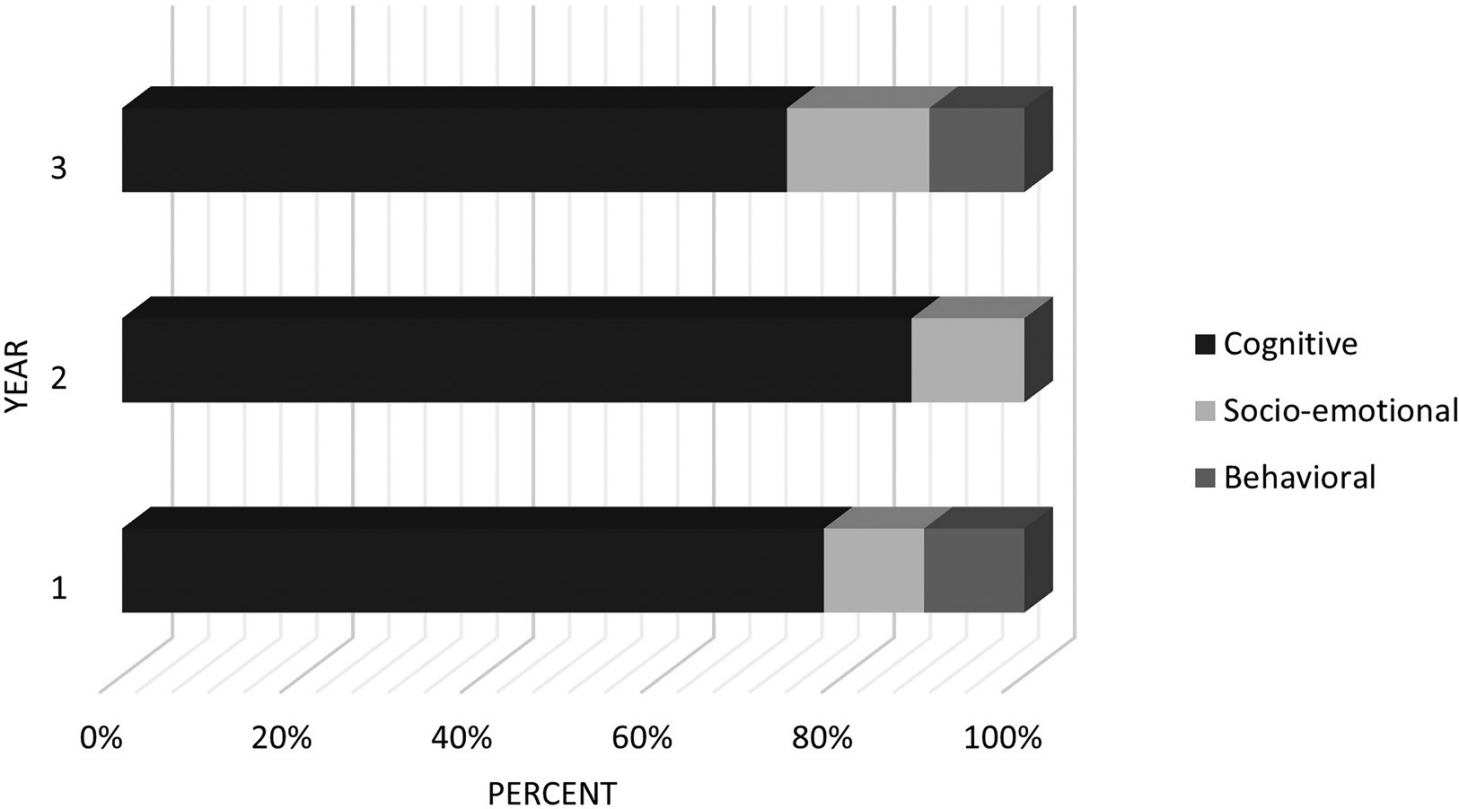
Overview of sustainability learning dimension referring each year.

A larger part of the SD-ILOs, *n* = 28 (78%) was categorized to the cognitive learning dimension for sustainable development, and *n* = 5 (14%) to the socio-emotional learning dimension and *n* = 3 (8%) to the behavioral learning dimension. The same pattern of distribution for sustainability learning dimensions was seen for each year of the education (Figure 2).

#### Key competencies for sustainable development

**Figure 3.**
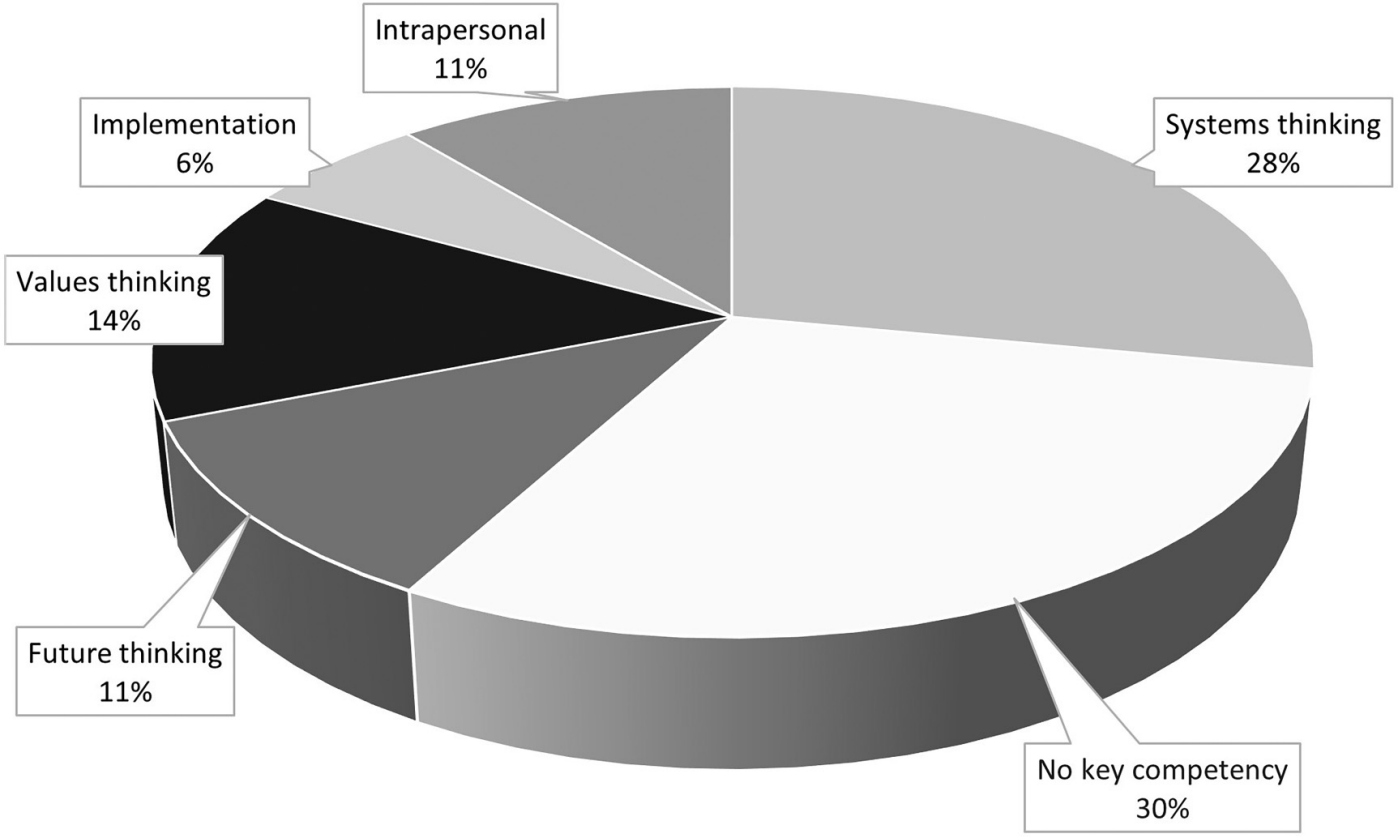
Sustainability-focused intended learning outcomes linked to key sustainability competencies.

More than two-thirds, *n* = 25 (70%) of the SD-ILOs were linked to a key competency for sustainable development (Figure 3). The most frequently identified competency was ‘systems-thinking’ *n* = 10 (28%). ‘Strategic-thinking’, ‘interpersonal’, and ‘integrated problem-solving’ competencies were not identified.

## Discussion

This study revealed that only 3% of the total number of ILOs in Swedish undergraduate physiotherapy education were sustainability-focused. To prepare future physiotherapists to deliver explicit actions for sustainable development, we believe this proportion must urgently increase. The uneven representation of SD-ILOs between the physiotherapy programs also calls for a national implementation strategy. The first steps should be to adhere to the Swedish Higher Education Act and for education authorities or policymakers within education to ensure that SD-ILOs are included. This would then be in line with the World Physiotherapy Europe Region statement on physiotherapy education, which clearly recommends that curricula for physiotherapy education should include perspectives of sustainable development.^34^

In light of the global threats to health such as climate change, loss of biodiversity, social inequality, and surging geopolitical conflicts, there is a compelling need for a paradigm shift within physiotherapy education. Mindful educational choices while developing SD-ILOs must be emphasized. It was interesting to learn that half of the SD-ILOs addressed sustainable development as content and half as a condition ie a topic or a perspective. Whether or not this is related to didactic choices remains unanswered, and neither of them could at this point be seen as either right or wrong since there are multiple ways of conceptualizing sustainable development.^35,36^ However, different focuses in SD-ILOs such as definitions of sustainable development, implementation of sustainable development strategies, or sustainable development as a discourse^7^ will foster critical thinking, creativity, and transformation in different ways. Also, addressing sustainable development as a content or a condition can be seen in the light of communication *about,* or *for* sustainable development,^37^ where *for* sustainable development has a better chance to establish societal transformation and actions towards sustainable development.^38^ Sustainable development should thus be viewed as a process in which students are actively engaged in constructing to facilitate meaningful and deep learning, rather than as pre-determined behaviors that they passively learn about.^37^ The European qualification framework for first cycle also states that students should ‘demonstrate advanced skills, demonstrating mastery and innovation required to solve complex and unpredictable problems in a specialized field of work or study’.^39^ Based on this we argue that the physiotherapy community must reflect on how SD-ILOs should frame sustainable development within physiotherapy education.^40^

The analysis of SD-ILOs revealed a majority of ILOs within the cognitive learning dimension, while only a limited number allocated to the socio-emotional and behavioral dimensions. It has been found that within primary, lower, and upper secondary schools, teachers feel more confident teaching cognitive skills within sustainable development, and less confident and knowledgeable about socio-emotional perspectives and behavioral learning.^41^ Whether or not this is mirrored within higher education and if a sense of confidence in teaching sustainable development is affecting the development of SD-ILOs should be further explored. However, our result is in line with other higher education institutions for example in the US where most SD-ILOs have been targeted within cognitive domain learning.^42^ Most SD-ILOs within physiotherapy undergraduate education in Sweden were also linked to either the cognitive processes of ‘understanding’ or ‘analyzing’. This highlights the need to further elaborate on cognitive processes, such as ‘applying’ and ‘creating’ to ensure physiotherapy students are prepared to make a difference in the real world. It is crucial to acknowledge that the nature of ESD must extend beyond the cognitive dimension and that reliance on theoretical knowledge alone is insufficient for fostering competence for sustainable development or contributing meaningfully to the attainment of the SDGs.^43^ Students require confidence not just in their knowledge about the issue, but also in their ability to exert positive influence to bring about change, including a drive for sustainable development as well as a commitment to finding solutions and action possibilities.^44^ We thus argue for the need to embrace all three dimensions of learning for sustainable development including learning activities that require complex cognitive learning processes in present and future physiotherapy undergraduate education. This is not only in line with UNESCO's recommendations^45^ but also aligns with the interconnected nature of the world, where deliberate action is needed from healthcare professionals to safeguard sustainable health.^46^

One-third of the SD-ILOs lacked clear connections to key competencies for sustainability, and the scarcity of SD-ILOs tied to key competencies such as values thinking, and intrapersonal competency suggests a gap in the current approach to ESD within physiotherapy curricula. Values thinking has been described as the lead competency through its ability to clarify and offer normative guidance in all other competencies, but it is feasible that intrapersonal competency likewise connects with the other competencies through its motivational and attitudinal elements.^24^ It has also been argued that intrapersonal competency might be an important element to include for fruitful integration of the cognitive, socio-emotional, and behavioral learning dimensions in ESD.^47^ Thus, the Swedish undergraduate physiotherapy education could increase its teaching and learning activities within the socio-emotional and behavioral learning dimensions, by for example prioritizing SD-ILOs that include affective capabilities and mindsets.^48^ Further, only two SD-ILOs were described as related to the more hands-on competency: implementation. For physiotherapy students to be able to act in relation to sustainable development, they must be provided the opportunity to actively apply a sustainability solution within a specific context. By doing so, students can gain firsthand experience and develop practical skills in implementing sustainable practices. Almost one-third of the SD-ILOs had the key competency ‘systems-thinking’ in focus, and this might reflect that systems thinking is at the core of physiotherapy practice.^49,50^ However, the concept of systems thinking can vary among health professionals^51^ and may also encompass varying degrees of a holistic view of sustainability including non-western knowledge along with other underlying drivers that uphold sustainability.^52,53^ It would therefore be interesting to further explore how teaching and learning activities related to systems thinking for sustainable development are used within physiotherapy education. The dearth of some key competencies such as strategic thinking and interpersonal competency also underscores the importance of working towards supporting students to develop a sense of agency for disruptive actions toward sustainable practices, through encouraging for example entrepreneurial attitudes, social awareness, professional boundary crossing, and collaboration.^54^

Some might argue that sustainable development through the third Sustainable Development Goal, about promoting healthy lifestyles, preventive measures, and modern efficient health care for everyone,^4^ is in line with the physiotherapy subject topic itself. However, the field of physiotherapy and health is intricately linked to almost all other SDGs, and physiotherapists as well as other health professionals, must therefore be prepared to contribute meaningfully to sustainable healthcare practices by including all three domains of sustainable development.^14^ This study does not provide answers to whether there is an equal focus in all these three domains within physiotherapy education in Sweden or if one dimension receives more attention in the SD-ILOs. That being said, it is worth noting that World Physiotherapy points out that physiotherapists need to be aware of the interconnections between climate change, sustainable development, and global health, and further emphasizes that physiotherapist undergraduate education should equip students with necessary knowledge, skills, and attitudes to support sustainable physiotherapy practices.^55^ For example, the climate injustice against people with disabilities has now received attention as climate change hazards worsen the vulnerabilities of people with disabilities, directly affecting their health.^56^ The potential impact of climate change on all other sustainable development goals is hence an urgent and major concern.^57^ Thus, moving away from the approach where sustainable development is viewed as the intersection of the three dimensions of sustainability, we argue for a future physiotherapy curriculum that drives sustainable development within planetary boundaries, based on the nested dependency model where economies and societies are seen as embedded parts of the biosphere.^58^ Consequently, in order to provide sustainable healthcare, physiotherapists need to understand the value of ecosystems and the anthropogenic threats to human and planetary health, and also learn how to reduce the negative environmental impact of healthcare.^59^

Lastly, the integration of sustainable development into physiotherapy education further necessitates a comprehensive exploration beyond the confines of syllabus adjustments. As we envision the future, not only is it essential to explore and implement changes in ILOs, but also to transform how we teach and learn for sustainable development.^60^ The current study did not explore the constructive alignment of ESD within physiotherapy, which should be considered as a topic for future research. Sustainable development is not just something to implement into a physiotherapy curriculum quickly and unreflective as a cosmetic change or only a brief bolt-on the course content. Rather, implementing sustainable development stresses a thoughtful build-in structure with a reformative delivery of the curriculum itself.^18,61^ Thus, a whole system re-design is needed, where values and norms within physiotherapy education as well as whole learning systems, structures, and cultures within HEs are challenged in order to organize ESD differently.^62^ Broadening the physiotherapy focus and rethinking who we serve and how might therefore lead to a way out of the ‘business as usual’^63^ at this critical point in time where transformative change is urgently needed. Moving forward, it thus becomes evident that developing effective ESD within physiotherapy education also requires an understanding of for example educators’ perceived competence in ESD and their educational choices made in terms of didactics for sustainability.

### Limitations

The study acknowledges that there are limitations in its methodology during the analysis of the syllabuses. The focus of this study was solely on the ILOs within local programs and course syllabuses at a given time. We have not delved into the course content or the literature related to sustainable development in the documents or through communication with course leaders. This limited scope could potentially lead to insufficient information such as the number of credits or hours spent on the topic, or how teachers’ understanding of the word sustainable development transfers into meaningful learning. Notably, physiotherapy program syllabuses in Sweden vary significantly in their level of detail, and thus our comparison focused exclusively on ILOs for consistency. Also, ILOs are measurable statements that articulate clearly what students should know, be able to do, or value as a result of taking a course which makes the exploration in this study relevant. The key competencies for sustainable development were also analyzed by studying ILOs, which is a fairly unexplored way of describing these competencies. However, there is no universally accepted way of assessing key competencies^64,65^ and therefore this study provides an important first step in the process of developing relevant teaching and learning strategies for these key competencies within physiotherapy.

## Conclusion

Only 3% of ILOs in Swedish undergraduate physiotherapy education were dedicated to sustainable development. Moreover, there was an unequal national distribution, underscoring the pressing demand for rapid and creative initiatives aimed at improving education for sustainable development within physiotherapy. There is also a need for broader pedagogical approaches that encompass not just the cognitive dimension of learning for sustainable development, but also socio-emotional and behavioral dimensions as well as more complex cognitive learning processes. The current lack of certain key competencies for sustainability further underscores the necessity to enhance physiotherapy curricula with action-oriented learning to develop powerful future sustainability agency within healthcare and the public health arena.

## Supplemental Material

sj-docx-1-mde-10.1177_23821205241260599 - Supplemental material for Time to Rethink Intended Learning Outcomes for Sustainable Development? A Qualitative Exploration and Reflection of Course Syllabuses in Swedish Undergraduate Physiotherapy Education Supplemental material, sj-docx-1-mde-10.1177_23821205241260599 for Time to Rethink Intended Learning Outcomes for Sustainable Development? A Qualitative Exploration and Reflection of Course Syllabuses in Swedish Undergraduate Physiotherapy Education by Emma Swärdh, Nina Brodin, Anna Pettersson and Annie Palstam in Journal of Medical Education and Curricular Development

## Notes

**Correction (June 2024):** Article updated to correct reference 55.
