# Supplementary material for: Time to Rethink Intended Learning Outcomes for Sustainable Development? A Qualitative Exploration and Reflection of Course Syllabuses in Swedish Undergraduate Physiotherapy Education
Source: J Med Educ Curric Dev. 2024 Jun 5;11:23821205241260599. doi: 10.1177/23821205241260599 (PMC11292048; doi:10.1177/23821205241260599)
Supplement: sj-docx-1-mde-10.1177_23821205241260599 - Supplemental material for Time to Rethink Intended Learning Outcomes for Sustainable Development? A Qualitative Exploration and Reflection of Course Syllabuses in Swedish Undergraduate Physiotherapy Education [file sj-docx-1-mde-10.1177_23821205241260599.docx]

**Supplementary Table: Checklist standards for reporting qualitative research (SRQR)**

| **Nr.** | **Item** | **Part of the manuscript where the information can be found** | **Manuscript page numbers*** |
| --- | --- | --- | --- |
| S1 | Title  Concise description of the nature and topic of the study identifying the study as qualitative or indicating the approach (e.g., ethnography, grounded theory) or data collection methods (e.g., interview, focus group) is recommended | Title | Title page, 1 |
| S2 | Abstract  Summary of key elements of the study using the abstract format of the intended publication; typically includes objective, methods, results, and  conclusions | Abstract | 3 |
| S3 | Problem formulation  Description and significance of the problem/phenomenon studied; review of relevant theory and empirical work; problem statement | Introduction | 4-6 |
| S4 | Purpose or research questions  Purpose of the study and specific objectives or questions | Introduction | 6-7 |
| S5 | Qualitative approach or research design  Qualitative approach (e.g., ethnography, grounded theory, case study, phenomenology, narrative research) and guiding theory if appropriate; identifying the research paradigm (e.g., positivist, constructivist/interpretivist) is also recommended | Research design  Data analysis | 7-8 |
| S6 | Researcher characteristics and reflexivity  Researchers’ characteristics that may influence the research, including personal attributes, qualifications/experience, relationship with participants, assumptions, or presuppositions; potential or actual interaction between researchers’ characteristics and the research  questions, approach, methods, results, or transferability | Data analysis | 8 |
| S7 | Context  Setting/site and salient contextual factors | Context and data collection | 7 |
| S8 | Sample strategy  How and why research participants, documents, or events were selected; criteria for deciding when no further sampling was necessary (e.g., sampling saturation) | Context and data collection | 7 |
| S9 | Ethical issues pertaining to human subjects  Documentation of approval by an appropriate ethics review board and participant consent, or explanation for lack thereof; other confidentiality and data security issues | Ethics | 8 |
| S10 | Data collection methods  Types of data collected; details of data collection procedures including (as appropriate) start and stop dates of data collection and analysis, iterative process, triangulation of sources/methods, and modification of procedures in response to evolving study findings | Context and data collection | 7 |
| S11 | Data collection instruments and technologies  Description of instruments (e.g., interview guides, questionnaires) and devices (e.g., audio recorders) used for data collection; if/how the instrument(s) changed over the course of the study | Theoretical framework for the deductive approach  Table 2 | 9-11 |
| S12 | Units of study  Number and relevant characteristics of participants, documents, or events included in the study; level of participation (could be reported in results) | Context and data collection  Data analysis  Results | - 1. & 11 |
| S13 | Data processing  Methods for processing data prior to and during  analysis, including transcription, data entry, data  management and security, verification of data  integrity, data coding, and anonymization /  deidentification of excerpts | Data analysis  Table 1 | 8 |
| S14 | Data analysis  Process by which inferences, themes, etc., were identified and developed, including researchers involved in data analysis; usually references a specific paradigm or approach | Data analysis  Table 1  Theoretical framework for the deductive approach | 8-11 |
| S15 | Techniques to enhance trustworthiness  Techniques to enhance trustworthiness and credibility of data analysis (e.g., member checking, audit trail, triangulation) | Data analysis  Table 1 | 8 |
| S16 | Synthesis and interpretation  Main findings (e.g., interpretations, inferences, and themes); might include development of a theory or model, or integration with prior research or theory | Result | 11-13 |
| S17 | Links to empirical data  Evidence (e.g., quotes, field notes, text excerpts, photographs) to substantiate analytic findings | Result  Table 3 & 4  Figure 1, 2 & 3 | 11-13 |
| S18 | Integration with prior work, implications, transferability, and contribution(s) to the field  Short summary of main findings; explanation of how findings and conclusions connect to, support,  elaborate on, or challenge conclusions of earlier scholarship; discussion of scope of application/generalizability; identification of unique contribution(s) to scholarship in a discipline or field | Discussion | 13-19 |
| S19 | Limitations  Trustworthiness and limitations of findings | Discussion | 19 |
| S20 | Conflict of interest  Potential sources of influence or perceived influence on study conduct and conclusions; how these were managed | Declaration of interest | Title page, 2 |
| S21 | Funding  Sources of funding and other support; role of funders in data collection, interpretation, and reporting | Funding | Title page, 2 |

*) all page numbers refer to the pages in the submitted manuscript file to the journal
